# Prevalence of hepatitis B virus infection among pregnant women in the mountainous regions of southern China: A retrospective single‐center study

**DOI:** 10.1002/jcla.24837

**Published:** 2023-01-05

**Authors:** Qiaoting Deng, Lifang Lin, Wei Guo, Xunwei Deng, Qunji Zhang, Jingyuan Hou

**Affiliations:** ^1^ Research and Experimental Center Meizhou People's Hospital Meizhou China; ^2^ Guangdong Provincial Key Laboratory of Precision Medicine and Clinical Translational Research of Hakka Population Meizhou China; ^3^ Guangdong Provincial Engineering and Technological Research Center for Clinical Molecular Diagnosis and Antibody Drugs Meizhou China; ^4^ Prenatal Diagnosis Center Meizhou People's Hospital Meizhou China

**Keywords:** Hakka, hepatitis B surface antigen, hepatitis B virus, pregnant women, southern China

## Abstract

**Background:**

Hepatitis B virus (HBV) infection remains a major public health issue worldwide. Moreover, its prevalence varies significantly in different geographic areas of China. The current study aimed to assess the prevalence of HBV infection among Hakka pregnant women in Meizhou, a remote mountainous region in southern China.

**Methods:**

This research was performed between January 2015 and December 2020. In total, 16,727 pregnant women receiving antenatal care at Meizhou People's Hospital were included in the analysis. All pregnant women were screened for serum HBV markers.

**Results:**

The prevalence rates of hepatitis B surface antigen (HBsAg) and hepatitis B surface antibody positivity among the participants were 11.74% (*n* = 1964) and 48.00% (*n* = 8029), respectively. The overall prevalence rates of susceptibility to infection, HBV immunity, previous/occult infection, inactive HBsAg carrier, and active infection were 36.16%, 33.61%, 16.94%, 8.11%, and 2.30%, respectively. According to age distribution, the prevalence rate of HBsAg positivity elevated concomitantly with increasing age (*p* < 0.001). From 2015 to 2020, the prevalence rate of HBsAg positivity decreased from 14.50% to 8.19% and that of hepatitis B pre‐core antigen positivity from 4.42% to 2.31%. In addition, pregnant women with HBsAg‐positive status were more likely to present with gestational diabetes, thrombocytopenia, and anemia than those with HBsAg‐negative status.

**Conclusion:**

The HBV infection rate remains high among pregnant women in the indigenous Hakka population in southern China. To prevent vertical transmission, cautious surveillance of maternal HBV infection status should be considered in Hakka pregnant women in Meizhou.

## Graphical abstract

This study aimed to assess the prevalence of HBV infection among Hakka pregnant women in Meizhou, a remote mountainous region in southern China. The results showed that the prevalence rates of HBsAg and HBsAb positivity among pregnant women were 11.74% and 48.00%, respectively. According to age distribution, the prevalence rate of HBsAg positivity elevated concomitantly with increasing age. From 2015 to 2020, the prevalence rate of HBsAg positivity decreased from 14.50% to 8.19%, and that of HBcAg positivity from 4.42% to 2.31%. In addition, HBsAg positive pregnant women were more likely to experience gestational diabetes, thrombocytopenia, and anemia than HBsAg negative pregnant women.

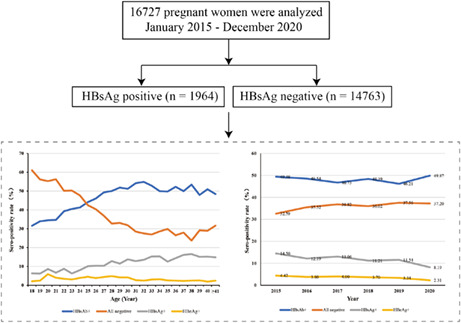

## 1 INTRODUCTION

Hepatitis B virus (HBV) infection remains a major public health issue posing a substantial socioeconomic burden. Globally, approximately 2 billion people have serologic evidence of previous or current infection with HBV, and more than 257 million have chronic HBV infection.^1^ Importantly, patients with HBV who are chronic carriers are at higher risk of progression to infection‐related long‐term sequelae such as chronic hepatitis, liver cirrhosis, hepatocellular carcinoma, and even death.^2^ In addition, the global prevalence of HBV infection varies in terms of geographic region, and it is highly endemic in resource‐limited countries, particularly those in East Asia, Sub‐Saharan Africa, and Western Pacific regions.^3^ In 2016, the World Health Organization committed to eliminating hepatitis B, a major public health threat, by 2030.^4^

HBV is transmitted between individuals via percutaneous or mucosal contact with contaminated blood and other body fluids. In highly endemic areas, the predominant route of HBV transmission is mother‐to‐child transmission (MTCT), accounting for approximately 90% of the global prevalence. Further, approximately 4.5 million women with chronic HBV infection give birth annually.^5^ Generally, infants infected with HBV have a 80%–90% risk of progression to a chronic disease state, which further develops to liver cirrhosis and hepatocellular carcinoma in young adulthood.^6^, ^7^, ^8^ By contrast, pregnant women with HBV are more likely to present with maternal and neonatal complications including preeclampsia, placenta previa, preterm delivery, placental separation, and gestational diabetes mellitus.^9^, ^10^, ^11^

Despite the existence of vaccines, HBV infection remains a major obstacle to public health in China.^12^ More than 86 million people are living with chronic HBV infection, and those with infection account for one‐third of the infected population worldwide.^13^ The overall efficacy rate of combined hepatitis B vaccine and hepatitis B immunoglobulin when used as a preventive strategy ranges from 90% to 95%. However, MTCT still occurs after passive‐active immunization, and it has become the major route of HBV infection among Chinese.^14^ HBV infection among women of reproductive age and pregnant women is endemic with regional variations.^15^ China has a large population, and it has been implementing the national two‐child policy since 2016. Therefore, antenatal screening for HBV serologic markers during pregnancy is significantly important in preventing HBV MTCT.

The economic and time costs have traditionally been a barrier that restricts follow‐up treatment of MTCT in remote and mountainous areas in China.^16^ Therefore, several high‐risk infants born to mothers positive to hepatitis B surface antigen (HBsAg) do not receive timely human hepatitis B immune globulin and the first vaccine dose. The prevalence of HBV infection remains high due to insufficient coverage rates of vaccination and limitations in terms of other preventive measures.^17^ Hence, studies about HBV infection among pregnant women may increase knowledge about the infection, thereby possibly contributing to transmission reduction. There are numerous epidemiological studies about HBV among pregnant women in China.^18^, ^19^ Meizhou City is a remote mountainous region located in the northeast of Guangdong Province in Southern China, with an area of 15,876 square kilometers and a population of 5.43 million. It remains one of the most undeveloped regions in China due to its mountainous terrains coupled with a sparsely distributed population. Hakka is an intriguing branch of the Han nationality with its own distinct Hakka dialect, lifestyle, culture, and architecture.^20^ Approximately 95% of inhabitants in the Meizhou region are of Hakka ethnicity. However, to date, there is no study about the prevalence of HBV infections among pregnant women in the study area, where most indigenous Hakka people settled.

The current retrospective study aimed to explore the prevalence of HBV infection among Hakka pregnant women in southern China. The results could provide updated data about the effective implementation of better vaccination programs and preventive strategies against HBV in southern China.

## 2 MATERIALS AND METHODS

### 2.1 Study population

A retrospective, single‐center study aimed to evaluate the prevalence of HBsAg among pregnant women attending antenatal clinics at Meizhou People's Hospital from January 2015 to December 2020. All pregnant women who visited this hospital underwent routine screening for HBV infection by performing blood tests during their initial prenatal visit. Data were retrospectively collected from the electronic database of Meizhou People's Hospital. Pregnant women without data about demographic characteristics and laboratory examination results and those with incomplete information about five HBV serology markers were excluded from the study. The research was performed in accordance with the ethical guidelines outlined in the 1975 Declaration of Helsinki. The current research was approved by the Ethics Committee of Meizhou People's Hospital. A written informed consent was obtained from all participants before the study initiation. All participants were self‐identified as indigenous people.

### 2.2 Sample collection and testing

In total, 5 ml of blood samples was collected aseptically and centrifuged to separate the serum within 3 h, and all serum specimens were evaluated within 24 h. The HBsAg, hepatitis B surface antibody (HBsAb), hepatitis B pre‐core antigen (HBeAg), hepatitis B pre‐core antibody (HBeAb), and hepatitis B core antibody (HBcAb) levels were evaluated using the Abbott i2000sr automatic immunoassay analyzer (Abbott Company, the USA). The markers of liver and kidney function were determined using AU5800 Automatic Analyzer (Beckman Coulter, Galway, Ireland). Complete blood count was evaluated using BC6600 plus Analyzer (Beckman Coulter, Galway, Ireland).

### 2.3 Definition

As shown in Table 1, based on five serological markers (HBsAg, HBsAb, HBeAg, HBeAb, and HBcAb) of HBV infection, different HBV statuses, including susceptibility to infection, HBV immunity, previous/occult HBV infection, inactive HBsAg carrier, and active HBV infection, were defined.

**TABLE 1:** Different statuses of HBV infection based on serological markers

HBV serological parameter					
HBsAg	HBsAb	HBeAg	HBeAb	HBcAb	Interpretation
−	−	−	−	−	Susceptible to infection
−	+	−	−	−	HBV immunity
−	+/−	−	+/−	+	Previous/occult infection
+	−	−	+/−	+	Inactive HBsAg carrier
+	−	+	−	+/−	Active infection

Abbreviations: HBsAg, hepatitis B surface antigen; HBsAb, hepatitis B surface antibody; HBeAg: hepatitis B e antigen; HBeAb: hepatitis B e antibody; HBcAb, hepatitis B core antibody.

### 2.4 Statistical analysis

All statistical analyses were performed using the Statistical Package for the Social Sciences software version 21.0 (IBM Inc., the USA). Continuous variables were presented as means ± standard deviations and categorical variables as numbers and percentages. Continuous variables were compared using the *t*‐test for two groups or analysis of variance for more than two groups. Meanwhile, the chi‐square test was utilized to analyze and compare categorical data. A *p*‐value of <0.05 was considered statistically significant.

## 3 RESULTS

**FIGURE 1:**
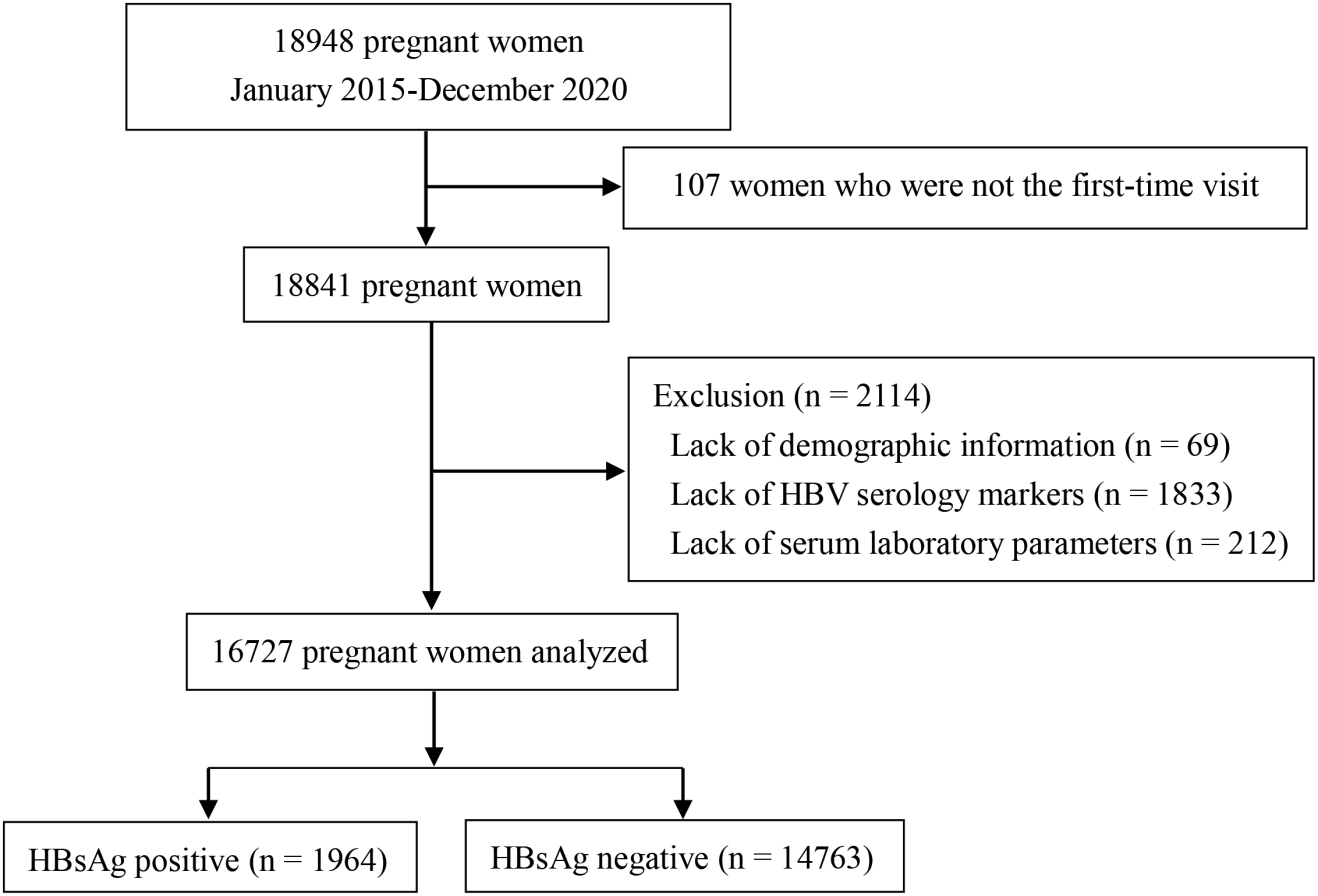
Study flow diagram.

Between January 2015 and December 2020, all pregnant women admitted to the antenatal units were screened. Among them, 2221 were excluded from the study. Figure 1 shows the study flow diagram. Therefore, 16,727 pregnant women were finally included in the analysis. The average age of the participants was 29.1 ± 5.3 years. Table 2 depicts the clinical characteristics of pregnant women. The participants were divided into the HBsAg‐positive and HBsAg‐negative groups. In total, 1964 pregnant women were HBsAg‐positive, with a prevalence rate of 11.74%. The HBsAg‐positive and HBsAg‐negative groups significantly differed in terms of age and marital status (*p* < 0.001). The HBsAg carriers were more likely to present with gestational diabetes (*p* = 0.011), thrombocytopenia (*p* < 0.001), and anemia (*p* = 0.004). Subsequently, the clinical characteristics of pregnant women were analyzed by HBV screening status. As shown in Table 3, the HBV status of pregnant women in the five different groups was as follows: susceptible to infection (36.16%, *n* = 6048), HBV immunity (33.61%, *n* = 5622), previous/occult infection (16.94%, *n* = 2834), inactive HBsAg carrier (8.11%, *n* = 1357), and active infection (2.30%, *n* = 384). Overall, the HBV status was significantly associated with marital status (*p* < 0.001), gestational diabetes (*p* < 0.001), preeclampsia (*p* = 0.022), thrombocytopenia (*p* = 0.004), and anemia (*p* = 0.015). However, the incidence of acute fatty liver of pregnancy (*p* = 0.594) and syphilis infection (*p* = 0.160) did not significantly differ among the groups.

**TABLE 2:** Demographics and clinical characteristics of pregnant women

	Total *n* (%)	HBsAg‐positive *n* (%)	HBsAg‐negative *n* (%)	*p*
Number	16,727	1964	14,763	
Age group				
<25 years	3162 (18.90)	234 (11.91)	2928 (19.83)	<0.001
25–35 years	11,391 (68.10)	1397 (71.13)	9994 (67.70)	
>35 years	2174 (13.00)	333 (16.96)	1841 (12.47)	
Marital status				
Single or Divorced	521 (3.11)	33 (1.68)	488 (3.31)	<0.001
Married	16,206 (96.89)	1931 (98.32)	14,275 (96.69)	
Gestational diabetes	3334 (19.93)	434 (22.10)	2900 (19.64)	0.011
Preeclampsia	978 (5.85)	118 (6.01)	860 (5.83)	0.746
Thrombocytopenia	250 (1.49)	51 (2.60)	199 (1.35)	<0.001
Anemia	3361 (20.09)	443 (22.56)	2918 (19.77)	0.004
Acute fatty liver of pregnancy	76 (0.45)	7 (0.36)	69 (0.47)	0.492
Syphilis infection	105 (0.63)	11 (0.56)	94 (0.64)	0.686

**TABLE 3:** HBV status of pregnant women

	Susceptible to infection, *n* (%)	HBV immunity, *n* (%)	Previous/occult infection, *n* (%)	Inactive HBsAg carrier, *n* (%)	Active infection, *n* (%)	*p*
Number	6048 (36.16)	5622 (33.61)	2834 (16.94)	1357 (8.11)	384 (2.30)	
Marital status						
Single or Divorced	279 (4.61)	162 (2.88)	42 (1.48)	16 (1.18)	14 (3.65)	<0.001
Married	5769 (95.39)	5460 (97.12)	2792 (98.52)	1341 (98.82)	370 (96.35)	
Gestational diabetes	1122 (18.55)	1099 (19.55)	624 (22.02)	315 (23.21)	79 (20.57)	<0.001
Preeclampsia	398 (6.58)	294 (5.23)	153 (5.40)	79 (5.82)	19 (4.95)	0.022
Thrombocytopenia	89 (1.47)	70 (1.25)	36 (1.27)	30 (2.21)	12 (3.13)	0.004
Anemia	1234 (20.40)	1072 (19.07)	552 (19.48)	313 (23.07)	81 (21.09)	0.015
Acute fatty liver of pregnancy	30 (0.50)	22 (0.39)	17 (0.60)	4 (0.29)	2 (0.52)	0.594
Syphilis infection	44 (0.73)	27 (0.48)	22 (0.78)	4 (0.29)	3 (0.78)	0.160

The association between biochemical parameters and different HBV infection statuses of pregnant women is summarized in Table 4. Pregnant women who were inactive HBsAg carriers and those with active infection had significantly higher serum glutamic‐pyruvic transaminase, glutamic oxaloacetic transaminase, total bile acid, total bilirubin, direct bilirubin, and creatinine levels than those with susceptibility to infection, HBV immunity, and previous/occult infection. In addition, pregnant women who were inactive HBsAg carriers and those with active infection had significantly lower albumin, prealbumin, uric acid, and hemoglobin levels and platelet count. Laboratory parameters, including alkaline phosphatase, glutamyl transpeptidase, total protein, and globulin levels, did not differ significantly between groups. Table 4 shows the data in detail.

**TABLE 4:** Association between biochemical parameters and different HBV infection statuses in pregnant women

	Susceptible to infection	HBV immunity	Previous/occult infection	Inactive HBsAg carrier	Active infection	*p*
ALT (U/L)	15.00 ± 29.28	15.53 ± 28.55	14.24 ± 17.85	19.44 ± 38.69	34.46 ± 58.85	<0.001
AST (U/L)	20.60 ± 25.64	21.24 ± 29.06	20.16 ± 13.98	24.01 ± 30.41	31.66 ± 36.65	<0.001
ALP (U/L)	144.77 ± 83.00	147.31 ± 86.42	143.35 ± 82.85	143.19 ± 75.69	149.10 ± 93.67	0.168
GGT (U/L)	12.14 ± 13.46	11.92 ± 12.56	11.71 ± 12.27	11.24 ± 10.21	12.59 ± 11.71	0.115
Total bile acid (μmol/L)	3.34 ± 7.24	3.06 ± 6.36	2.88 ± 4.87	4.08 ± 7.45	5.16 ± 15.27	<0.001
Total bilirubin (μmol/L)	9.13 ± 6.80	9.18 ± 5.87	9.18 ± 4.58	9.53 ± 4.89	10.31 ± 11.60	0.002
Direct bilirubin (μmol/L)	2.20 ± 3.68	2.12 ± 2.99	2.10 ± 1.74	2.22 ± 2.01	2.59 ± 6.32	0.036
Total protein (g/L)	61.33 ± 6.70	61.57 ± 6.47	61.56 ± 6.60	61.05 ± 6.63	61.20 ± 7.76	0.050
Albumin (g/L)	33.97 ± 4.48	34.20 ± 4.28	34.03 ± 4.48	33.71 ± 4.56	33.79 ± 4.97	0.002
Globulin (g/L)	27.36 ± 3.98	27.37 ± 3.83	27.53 ± 3.82	27.35 ± 3.60	27.41 ± 4.31	0.378
Prealbumin (mg/L)	176.02 ± 43.42	177.59 ± 42.25	179.25 ± 45.17	164.47 ± 41.63	154.92 ± 42.46	<0.001
Uric acid (μmol/L)	303.37 ± 94.32	300.13 ± 91.51	302.73 ± 91.35	293.02 ± 88.25	286.62 ± 89.89	<0.001
Creatinine (μmol/L)	64.64 ± 15.85	65.46 ± 19.03	64.29 ± 17.02	66.78 ± 15.55	67.27 ± 12.34	<0.001
Leukocyte (10^9^/L)	10.86 ± 3.64	10.73 ± 4.06	10.59 ± 3.52	10.45 ± 3.50	10.85 ± 4.39	0.001
Erythrocyte (10^12^/L)	4.07 ± 0.54	4.08 ± 0.53	4.06 ± 0.52	4.04 ± 0.55	4.06 ± 0.53	0.064
Hemoglobin (g/L)	114.20 ± 15.81	116.02 ± 15.39	115.39 ± 15.68	114.61 ± 15.44	113.86 ± 16.32	<0.001
Platelet (10^9^/L)	226.29 ± 64.87	222.57 ± 62.53	221.98 ± 62.41	210.58 ± 60.84	210.82 ± 65.74	<0.001

*Note*: Data were presented as mean value ± standard deviation.

Abbreviations: ALT, glutamic‐pyruvic transaminase; AST, glutamic oxalacetic transaminase; ALP, alkaline phosphatase; GGT, glutamyl transpeptidase.

**FIGURE 2:**
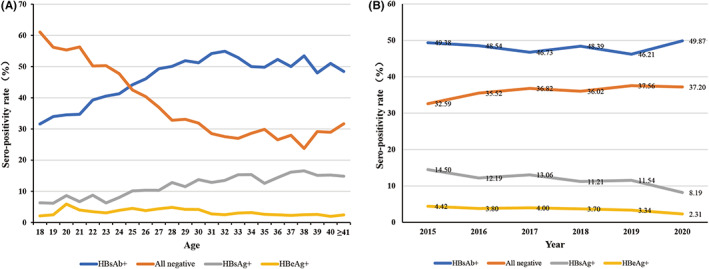
Serological markers of hepatitis B virus in 16,727 pregnant women according to different age (A) and year (B) groups.

Table 5 and Figure 2 show the prevalence of different HBV infection statuses between age and year groups. Approximately 48.00% and 3.59% of pregnant women were HBsAb^+^ and HBeAg^+^, respectively. Based on age distribution, the prevalence rate of HBsAg positivity, previous/occult infection, and inactive HBsAg carrier elevated concomitantly with increasing age (all *p* < 0.001). However, pregnant women had a significantly lower prevalence rate of susceptibility to infection and active infection concomitantly with increasing age (*p* < 0.001 and *p* = 0.003, respectively). Approximately 51.71% of pregnant women aged <25 years were negative for serological markers, and this rate was higher than that among pregnant women aged 25–35 (33.41%) years and those aged >35 (27.92%) years. From 2015 to 2020, the prevalence of susceptibility to infection among pregnant women slightly increased from 32.59% to 37.56% (*p* = 0.008). The prevalence of HBV immunity was stable at approximately 34% annually from 2015 to 2020, and the prevalence rate of HBsAb positivity constantly ranged from 46.21% to 49.87%. In addition, the prevalence rate of inactive HBsAg carrier and active infection were relatively decreasing from 2015 to 2020 (*p* < 0.001). The prevalence of HBsAg positivity decreased from 14.50% to 8.19% and that of HBeAg positivity from 4.42% to 2.31%.

**TABLE 5:** Prevalence of different HBV infection statuses according to age and year groups

Groups	Overall (*n* = 16,727)	Age, *n* (%)				Year, *n* (%)						*p*
		<25 (*n* = 3162)	25–35 (*n* = 11,391)	>35 (*n* = 2174)	*p*	2015 (*n* = 1924)	2016 (*n* = 2872)	2017 (*n* = 3178)	2018 (*n* = 3112)	2019 (*n* = 3259)	2020 (*n* = 2382)	
Susceptible to infection	6048 (36.16)	1635 (51.71)	3806 (33.41)	607 (27.92)	<0.001	627 (32.59)	1020 (35.52)	1170 (36.82)	1121 (36.02)	1224 (37.56)	886 (37.20)	0.008
HBV immunity	5622 (33.61)	982 (31.06)	3952 (34.69)	688 (31.65)	<0.001	666 (34.62)	993 (34.58)	1073 (33.76)	1038 (33.35)	1035 (31.76)	817 (34.30)	0.173
Previous/occult infection	2834 (16.94)	278 (8.79)	2061 (18.09)	495 (22.77)	<0.001	313 (16.27)	448 (15.60)	439 (13.81)	577 (18.54)	594 (18.23)	463 (19.44)	<0.001
Inactive HBsAg carrier	1357 (8.11)	117 (3.70)	962 (8.45)	278 (12.79)	<0.001	194 (10.08)	241 (8.39)	287 (9.03)	233 (7.49)	264 (8.10)	138 (5.79)	<0.001
Active infection	384 (2.30)	80 (2.53)	276 (2.42)	28 (1.29)	0.003	84 (4.37)	85 (2.96)	73 (2.30)	52 (1.67)	58 (1.78)	32 (1.34)	<0.001
HBsAg+	1964 (11.74)	234 (7.40)	1397 (12.26)	333 (15.32)	<0.001	279 (14.50)	350 (12.19)	415 (13.06)	349 (11.21)	376 (11.54)	195 (8.19)	<0.001
HBsAb+	8029 (48.00)	1212 (38.33)	5717 (50.19)	1100 (50.60)	<0.001	950 (49.38)	1394 (48.54)	1485 (46.73)	1506 (48.39)	1506 (46.21)	1188 (49.87)	0.041
HBeAg+	600 (3.59)	117 (3.70)	431 (3.78)	52 (2.39)	0.006	85 (4.42)	109 (3.80)	127 (4.00)	115 (3.70)	109 (3.34)	55 (2.31)	0.003

Abbreviations: HBsAg, hepatitis B surface antigen; HBsAb, hepatitis B surface antibody; HBeAg, hepatitis B e antigen.

We compared the prevalence of HBsAg positivity in pregnant women in different regions in China based on several HBV epidemiological studies between 2014 and 2021. Individual studies revealed a significant variation in terms of prevalence across geographical areas, as shown in Table 6.

**TABLE 6:** Prevalence rates of HBsAg positivity among pregnant women in different regions in China

Geographical Region	Province	Sample size	HBsAg positivity	Prevalence of HBsAg positivity	Study and Year
Northeast of China	Liaoning	14,314	441	3.08%	Sheng et al. 2018^24^
Northwest of China	Shaanxi	13,451	951	7.07%	Chen et al. 2021^10^
North of China	Tianjin	1829	69	3.77%	Kang et al. 2021^25^
North of China	Shanxi	10,311	505	4.90%	Zhang et al. 2014^26^
East of China	Zhejiang	19,500	1146	5.88%	Wu et al. 2020^23^
East of China	Anhui	3329	346	10.39%	Cai et al. 2019^19^
West of China	Xinjiang	10,284	456	4.43%	Zhang et al. 2014^26^
Southwest of China	Sichuan	22,374	948	4.24%	Tan et al. 2016^27^
Southwest of China	Yunnan	49,474	1624	3.28%	Sun et al. 2021^28^
Southeast of China	Fujian	85,190	9699	11.39%	Zhang et al. 2020^22^
Southeast of China	Guangdong	39,539	3039	7.69%	Yin et al. 2021^18^
South of China	Guangdong	16,727	1964	11.74%	The present study. 2020

## 4 DISCUSSION

Despite the provision of vaccines, HBV infection remains a major concern worldwide, particularly in developing countries.^1^ The significant progress in antiretroviral treatment caused a remarkable decline in morbidity and mortality associated with HBV infection. However, HBV can be easily transmitted from mother to child during delivery. Antiretroviral therapy for pregnant women reduces the incidence of MTCT and maternal and child mortality.^14^ Pregnant women should be screened promptly during their antenatal care visits, and proper treatment must be provided to decrease the rate of MTCT. Therefore, the current retrospective, single‐center study aimed to evaluate the HBV infection status of pregnant women in Meizhou, southern China. Results showed that the overall prevalence of HBsAg positivity was extremely high at 11.74% among pregnant women in the study area. Moreover, the prevalence of HBV infection increased with age and decreased continuously from 2015 to 2020. In addition, the following maternal adverse effects were observed: gestational diabetes, thrombocytopenia, and anemia. The current study provided critical data that can be used for assessing the impact of current prevention strategies and for planning vaccination and other preventive measures in Meizhou.

Currently, the global prevalence of HBV infection is highly heterogeneous.^21^ The classification of high endemic is defined as a prevalence rate of ≥8%. Previous studies have reported that the proportion of HBV carriers is relatively low at 0.1%–2.0% in the United States and Western Europe. However, it is high (up to 8%–20%) in Africa and most countries in Asia.^3^ HBV infection remains a significant public health issue in China, with prevalence varying significantly in different geographic areas, even if it is heterogeneous within a region among different studies. The current study showed that the prevalence rate of HBsAg positivity in the study area was 11.74%, which was similar to that in Fujian (11.39%)^22^ and Anhui (10.39%).^19^ However, it was higher than that in Shaanxi (7.07%),^10^ Guangdong (7.69%),^18^ and Zhejiang (5.88%).^23^ Similar to other reports, the prevalence of HBV infection was significantly lower in Liaoning (3.08%),^24^ Tianjin (3.77%),^25^ Shanxi (4.90%),^26^ Xinjiang (4.43%),^26^ Sichuan (4.24%),^27^ and Yunnan (3.28%).^28^ The prevalence rate of HBV infection differed among pregnant women in the provinces of China and at different periods. Moreover, recent national epidemiological data showed that the prevalence of HBV infection continuously declined among pregnant women in China. However, the regional pockets of relatively high HBV endemicity also remained in southern and southeastern China.^15^ These remarkable differences could be attributed to socioeconomic status, public health education, level of awareness, and infection prevention practices by the community.

HBV vaccines have been available since the early 1980 s. China is among the developing countries that integrated the hepatitis B vaccine for newborns into routine immunization in 1992. However, parents had to pay out of pocket.^12^ Therefore, infant vaccination coverage was uneven, which was relatively low in underdeveloped rural areas. In 2002, the Chinese government implemented the national immunization program, thereby making it free for infants. The universal HBV immunization policies and measures for childhood constantly improved over the last three decades.^14^ Consequently, the overall prevalence of HBV infection in China has decreased significantly from 9.8% in 1992 to 7.2% in 2006.^29^ For example, an epidemiological survey in Guangdong revealed that the prevalence of HBsAg among children aged 1–14 years decreased significantly from 19.9% in 1992 to 1.2% in 2013.^30^ Our research showed that the prevalence of HBV among pregnant women is high in Meizhou. Moreover, this further supports the notion that the prevalence of HBsAg was the highest in Eastern Guangdong and was low in the Pearl River Delta and western Guangdong regions. This may partly explain the rural economy lags behind the urban economy in China, along with health and education resources.^13^, ^29^ From a policy perspective point of view, the prevalence of HBV infection should be updated to support targeted immunization and health education and enhanced interventions for MTCT prevention.

HBsAg positivity represents active acute or chronic infections. Meanwhile, HBeAg indicates active replication and a high HBV infectivity.^31^, ^32^ HBeAg positivity among childbearing‐age women is a major determinant of perinatal HBV transmission. Previous studies have revealed that without any intervention, the frequency of HBV perinatal transmission can reach up to 90% in infants born to women who are HBsAg and HBeAg carriers.^33^ In this study, the positivity rates of HBsAg and HBeAg in pregnant women were 8.19%–14.50% and 2.31%–4.42%, respectively. In HBV endemic areas, HBV infections commonly occur perinatally, and they are observed in unvaccinated or inadequately vaccinated subgroups of children. Further, regular antenatal screening of pregnant women is not common and compulsory in Meizhou. This finding may reflect previous outbreaks affecting adult populations in the study area. The high infection rate might be attributed to poverty, lack of health education and knowledge about infection status, and poor awareness of HBV infection prevention among our study groups. In individuals who are immunocompetent, hepatitis B vaccines can have protective effects lasting for at least two decades.^34^ Nonetheless, anti‐HBs vaccination titers can wane with time and may decrease below protective levels. The prevalence of HBsAg in pregnant women increased with age, with the highest positivity rate in participants aged >35 years, followed by those aged 25–35 years. Meanwhile, participants aged >25 years had the lowest rates. In accordance with our findings, other studies showed that the seroprevalence of HBV infection increased with age in both pregnant women and the general population.^35^, ^36^ The effect of age may be correlated with the progressive loss of protective antibody levels against HBsAg over time. However, the seropositive rate of HBsAg or the prevalence of HBeAg decreased over time. Notably, as there was a continuous decrease in positivity rates, our study population was assumed to benefit from routine immunization against HBV infection that was rolled out to infants and neonates in China. Nevertheless, the high prevalence rate of HBV in our study population remains a significant concern. This emphasizes the need for continuous routine antenatal HBsAg assessment among pregnant women and the implementation of appropriate measures during childbirth to reduce transmission.

The association between HBV serological outcomes and maternal complications has been contrasting.^9^, ^28^ In our study, pregnant women positive for HBsAg were more likely to present with gestational diabetes. This result is consistent with that of other retrospective cohort studies conducted on the Chinese population.^10^, ^27^, ^37^ Moreover, this result has not been confirmed in Asian and American pregnant women.^11^, ^38^ Previous studies have shown that HBV infection does not affect the development of preeclampsia,^27^, ^39^ and our result is in accordance with this finding. However, other studies did not identify such an association or had contrasting results.^40^ Patients with elevated ALT levels before treatment had a higher incidence of post‐partum hepatitis flare.^41^, ^42^ Our results found that HBsAg‐positive participants, particularly those who are HBeAg carriers, had significantly high ALT levels. High antepartum ALT levels might indicate a greater immune response against HBV and might further lead to post‐partum hepatic flares.^43^ The current study showed that pregnant women with HBsAg‐positive status were more likely to present with anemia and thrombocytopenia. This is supported by evidence showing that chronic liver diseases are commonly associated with hematological abnormalities. A previous study has shown that the presence of anemia in patients with HBV infection could indicate alterations in liver function.^44^ A recent study revealed that pregnant women with HBV infection had a threefold increased risk for anemia compared with those with human immune virus‐positive status but without HBV infection.^45^ One large cohort study has found a strong association between HBV infection and incident thrombocytopenia.^46^ Early detection of hepatitis B virus infection before pregnancy should be considered in clinical practice, and cautious surveillance must be performed.

The current study had several limitations that should be acknowledged. That is, it was a single‐center, hospital‐based, retrospective study. Therefore, selection bias might have existed, and its capability of inferences was limited. Second, data about vaccination information, educational level, monthly income, and occupation among pregnant women were not collected. Third, follow‐up analysis of maternal and neonatal outcomes was not performed.

## 5 CONCLUSION

To the best of our knowledge, this is the first study that assessed the prevalence of HBV infection among Hakka pregnant women. Results showed that the seropositivity rate of HBsAg or the prevalence of HBeAg positivity among pregnant women decreased over time from 2015 to 2020 in Meizhou. Nevertheless, the prevalence rate of HBV infection among pregnant women remained high. Hence, cautious surveillance of maternal HBV infection status should be recommended, and immunization programs must be strengthened.

## FUNDING INFORMATION

This work was funded by Guangdong Provincial Key Laboratory of Precision Medicine and Clinical Translation Research of Hakka Population (2018B030322003), Science and Technology Program of Meizhou (2019B0202001).

## CONFLICT OF INTEREST

The authors declare no competing interests.

## Data Availability

Data are available upon request from the corresponding author.
